# Impact of the Host-Microbiome on Osteomyelitis Pathogenesis

**DOI:** 10.3389/fmolb.2021.702484

**Published:** 2021-08-09

**Authors:** Jun Chen, Ailin Xiong, Yuhao Ma, Chenghe Qin, Chun Loong Ho

**Affiliations:** ^1^ Department of Biomedical Engineering, Southern University of Science and Technology (SUSTech), Shenzhen, China; ^2^ Department of Orthopaedic Trauma, Guangdong Second Provincial General Hospital, Guangzhou, China

**Keywords:** microbiome, osteomyelitis, direct interaction, indirect interaction, pathogenesis

## Abstract

The microbiome is a collection of genomes from microbiota, including all microorganisms in a niche, through direct and indirect interactions with the host. Certain microorganisms can exist in areas conventionally considered to be sterile, such as the bone matrix. Osseous microbiota dysbiosis caused by host-microbiome perturbation or external infections may ultimately lead to osteomyelitis, a bone inflammatory disorder. Our review covers the current discoveries on the impact of host-microbiome on osteomyelitis and some common osseous diseases. Some studies suggest that the microbiotas from both osseous and non-osseous tissues (e.g., blood or gut) impact the pathogenicity of osteomyelitis and other osseous diseases (e.g., rheumatoid arthritis). We believe that this review will provide readers with a better understanding on the role of the microbiome to the host’s bone health.

## Introduction

Osteomyelitis is a bone inflammatory disease that usually results from microbial infections. In other instances, certain osteomyelitic cases are not caused by microbial infections and exhibit auto-inflammatory bone disorders, e.g., chronic nonbacterial osteomyelitis (Buch et al., 2019). The diverse forms of osteomyelitis are classified according to differentiating features, such as pathogenesis, duration of infection, localization, implant presence, anatomy, and co-morbidity (Zimmerli, 2015).

The recent development of microbiome research shows that the host microbiota interacts with the body to maintain a homeostatic balance or exacerbate the state of infection in the host. Microbiome, the collection of genomes from the microbiota, varies from the different niches in the host (e.g., oral cavity, skin, and gastrointestinal tract). While the microbiome of each niche is unique and separate, it can influence different remote areas of the host and the corresponding microbiome within that niche.

Given the tenacious nature of osteomyelitic infections, investigating the impacts of the host microbiota on osteomyelitis is critical to understanding the pathology, etiology, diagnosis, prevention, therapeutics, and prognosis of osteomyelitis. Osteomyelitis is correlated with the microbiota within the osteomyelitic bones and microbes in blood and other distant organs, including the gastrointestinal tract, via indirect interactions. Herein, we provide a review on the impact of the host-associated microbiome on osteomyelitis. This review would provide readers a better understanding of the role of the host microbiome and how the microbiome dysbiosis influences the host’s susceptibility to osteomyelitis. The review is divided into two main sections addressing direct and indirect microbiome-associated osteomyelitis.

## Osteomyelitis Directly Associated With Microbiome

Osteomyelitis is generally associated with microbial infections. These infectious microorganisms (Table 1) in osteomyelitic sites are commonly detected using cultivation-, microscope-, histology- and sequencing-based methods. The conventional cultivating methods mainly utilize aerobic and anaerobic conditions to cultivate microbes from osteomyelitic samples (Lavery et al., 1995; Abdulrazak et al., 2005; Van Asten et al., 2016). In contrast, the emergence of culturomics leverages multiple culture conditions combined with the rapid identification of microbes, rendering isolation of over 3,000 microbial colonies possible (Jneid et al., 2018). Nevertheless, the cultivation-based approaches are only capable of screening species that are culturable under the typical nutritional conditions, thus incapable of reflecting the actual microbial abundance and distribution. Additionally, culturomics requires laborious and time-consuming cultivating steps, limiting its practical applications to large sample sizes.

**TABLE 1:** Main microorganisms involved in osteomyelitis.

Main microbiota compositions	Location	Possible interacting mechanisms	References
Gram positive bacteria			
*Actinomyces* sp. (including *A. israelii*, *A. naeslundii*, *A. viscosus*)	DF/Jaw	Evidence suggests that *Actinomyces* influences the pathogenesis of chronic infection.	Gaetti-Jardim Júnior et al. (2010); Lesens et al. (2011); Goda et al. (2014); Van Asten et al. (2016)
*Anaerococcus* sp.	DF	NA	Cai et al. (2019); Johani et al. (2019)
*Bacillus* sp.	DF	NA	Lesens et al. (2011); Jneid et al. (2018)
*Corynebacterium* sp.	DF	NA	Abdulrazak et al. (2005); Lesens et al. (2011); Van Asten et al. (2016); Johani et al. (2019)
*Campylobacter rectus*	DF	NA	Gaetti-Jardim Júnior et al. (2010)
*Clostridium* sp.	DF	NA	Lesens et al. (2011)
*Cutibacterium acnes*	Jaw	Virulence factors of *C. acnes* (e.g., beta-lactamase) and genes encoding biofilm biosynthesis increases the chances of localization in osteomyelitic sites.	Park et al. (2017)
*Eikenella corrodens*	DF	NA	Gaetti-Jardim Júnior et al. (2010)
*Enterococcus* sp. (including *E. faecalis*)	DF/Jaw	NA	Lavery et al. (1995); Abdulrazak et al. (2005); Gaetti-Jardim Júnior et al. (2010); Jneid et al. (2018); Zou et al. (2020)
*Enterobacter cloacae*	DF	NA	Abdulrazak et al. (2005)
*Eubacterium* sp.	DF/Jaw	NA	Lesens et al. (2011); Goda et al. (2014)
*Filifactor alocis*	Jaw	NA	Goda et al. (2014)
*Finegoldia* sp. (including *F. magna*)	DF	NA	Abdulrazak et al. (2005); Wei et al. (2012); Van Asten et al. (2016); Johani et al. (2019)
*Helcococcus* sp.	DF	NA	Van Asten et al. (2016)
*Micrococcus* sp.	DF	NA	Lavery et al. (1995); Lesens et al. (2011); Jneid et al. (2018)
*Mogibacterium timidum*	Jaw	NA	Goda et al. (2014)
*Peptococcus* sp.	DF	NA	Lesens et al. (2011)
*Peptostreptococcus* sp.	DF	NA	Lesens et al. (2011)
*Propionibacterium* sp. (including *P. acnes*)		NA	Gaetti-Jardim Júnior et al. (2010); Lesens et al. (2011); Van Asten et al. (2016)
*Proteus* sp. (including *P. mirabilis* and *P. vulgaris*)	DF	*P. vulgaris* was positively correlated with the infection index, but the mechanistic investigation is limited.	Abdulrazak et al. (2005); Gaetti-Jardim Júnior et al. (2010); Cai et al. (2019); Zou et al. (2020)
*Parvimonas micra*	DF/Jaw	*P. micra* is predominantly found in periodontitis, gingivitis, and dental periapical abscesses, however their virulence factors remain unclear.	Gaetti-Jardim Júnior et al. (2010); Goda et al. (2014)
*Pseudoramibacter alactolyticus*	Jaw	NA	Goda et al. (2014)
*Staphylococcus aureus*	DF	*S. aureus* infects the host in several approaches (Figure 1). *S. aureus* can bind to the host tissues, leading to biofilm form that evades the host’s immune system.	Lavery et al. (1995); Abdulrazak et al. (2005); Gaetti-Jardim Júnior et al. (2010); Lesens et al. (2011); Van Asten et al. (2016); Jneid et al. (2018); Zou et al. (2020)
*Staphylococcus* others species	DF	These species are generally involved with skin and soft tissue infections, but the mechanism implicated in osteomyelitis is not clear yet.	Abdulrazak et al. (2005); Gaetti-Jardim Júnior et al. (2010); Lesens et al. (2011); Van Asten et al. (2016); Johani et al. (2019); Zou et al. (2020)
*Streptococcus* sp. (including *S. lugdunensis*, *S. epidermidis, S. pyogenes, S. castoreus*)	DF/Jaw	*Streptococci* are commonly found in hematogenous osteomyelitis, but the pathogenetic mechanism is not ascertained.	Jneid et al.; Lavery et al. (1995); Abdulrazak et al. (2005); Gaetti-Jardim Júnior et al. (2010); Lesens et al. (2011); Wei et al. (2012); Goda et al. (2014); Van Asten et al. (2016); Johani et al. (2019); Zou et al. (2020)
*Treponema* sp. (including *T. denticola* and *T. maltophilum*)	DF/Jaw	NA	Gaetti-Jardim Júnior et al. (2010); Goda et al. (2014)
*Veillonella parvula*	DF	NA	Cai et al. (2019)
Gram negative bacteria			
*Bacteroides* sp.	DF	NA	Abdulrazak et al. (2005); Lesens et al. (2011); Wei et al. (2012)
*Campylobacter* (including *C. gracilis*)	Jaw	NA	Goda et al. (2014)
*Citrobacter* (including *C. koseri*)	DF	NA	Lesens et al. (2011); Zou et al. (2020)
*Desulfomicrobium orale*	Jaw	NA	Goda et al. (2014)
*Dialister pneumosintes*	Jaw	NA	Goda et al. (2014)
*E. coli*	Jaw/DF	NA	Abdulrazak et al. (2005); Lesens et al. (2011); PaDhiary et al. (2013); Zou et al. (2020)
*Enterobacter* sp. (including *E. cloacae*)	DF	NA	Abdulrazak et al. (2005); Lesens et al. (2011); Van Asten et al. (2016); Jneid et al. (2018); Zou et al. (2020)
*Fusobacterium nucleatum*	DF	*F. nucleatum* may play a role in osteomyelitic pathogenesis, including biofilm formation.	Gaetti-Jardim Júnior et al. (2010); Lesens et al. (2011); Goda et al. (2014)
*Klebsiella* sp. (including *K. pneumoniae*)	DF	NA	Lesens et al. (2011); Cai et al. (2019); Zou et al. (2020)
*Morganella* sp. (including *M. morgani*)	DF	NA	Abdulrazak et al. (2005); Lesens et al. (2011)
*Neisseria* sp. (including *N. bacilliformis*)	Jaw	NA	Lesens et al. (2011); Goda et al. (2014)
*Odoribacter denticanis*	Jaw	NA	Goda et al. (2014)
*Phocaeicola abscessus*	Jaw	NA	Goda et al. (2014)
*Porphyromonas* (including *P. gingivalis* and *P. endodontalis*)	DF	*P. gingivalis* may synergistically form biofilms with *F. nucleatum* in osteomyelitic tissues.	Gaetti-Jardim Júnior et al. (2010); Wei et al. (2012); Goda et al. (2014); Van Asten et al. (2016); Johani et al. (2019)
*Prevotella* (including *P. intermedia, P. nigrescens*)	DF	*Prevotella* had positive correlation with the duration of diabetic foot infection, however the mode of action is poorly understood.	Gaetti-Jardim Júnior et al. (2010); Wei et al. (2012); Van Asten et al. (2016); Cai et al. (2019); Zou et al. (2020)
*Proteus species* (including *P. mirabilis* and *P. mirabilis*)	DF	NA	Abdulrazak et al. (2005); Lesens et al. (2011); Jneid, et al. (2018)
*Pseudomonas aeruginosa*	DF	NA	Abdulrazak et al. (2005); Lesens et al. (2011); Zou et al. (2020)
*Pseudomonas* other species	DF	NA	Lesens et al. (2011); Van Asten et al. (2016)
*Serratia marcescens*	DF	NA	Abdulrazak et al. (2005); Zou et al. (2020)
*Tannerella forsythia*	Jaw	*T. forsythia* may synergistically form biofilms with *F. nucleatum* in osteomyelitic tissues.	Goda et al. (2014)
*Veillonella* sp.	DF	NA	Lesens et al. (2011)

a Note: DF = diabetic foot; NA = mechanism is not provided in the reference.

High-throughput sequencing or the next generation sequencing (NGS) is a swifter and more accurate approach that overcomes the above shortcomings and is currently widely used to quickly and comprehensively investigate microbiome. Common NGS-based methods for studying osteomyelitis comprise the 16S rRNA gene sequencing and metagenome sequencing. The 16S rRNA gene sequencing generally studies the variable regions instead of the whole gene following PCR amplification. In contrast, metagenome sequencing analyzes all microorganisms’ complete genomes in a specimen and provides microbial identity at the species level. However, metagenomics cannot identify the microbial behaviors or the metabolic states (Cassir et al., 2016). Thus, metagenomic sequencing is often coupled to other molecular approaches, e.g., transcriptomics and proteomics, to acquire complete information on the actual functions of the microbiome.

Microscopic methods have also been employed to investigate the osteomyelitic microbiome, providing the exact microbial locations and other taggable biochemical information. These approaches include scanning electron microscopy (SEM) and peptide nucleic acid fluorescent *in situ* hybridization (PNA-FISH) coupled to confocal laser scanning microscopy (CLSM), e.g., a combinative utilization of them shows the predominance of coccoid microorganisms (Malone et al., 2019) and the biofilm formation (Johani et al., 2019) in osteomyelitic tissues.

These various approaches applied to study the microbiome have their respective advantages and disadvantages. The cultivation-based methods can provide physiological properties and assess the potential for virulence and antimicrobial resistance at the strain level. Molecular methods generally identify more microbes than the culture-based methods (Malone et al., 2019; Zou et al., 2020), and the highly efficient NGS can quickly and accurately provide microbial diversity and distribution (Lagier et al., 2015). The advantages of these methods complement each other limitations and are often performed in tandem for better characterization of the osteomyelitic microbiome. These approaches have been successfully used to study the direct microbiome-associated osteomyelitis of diabetic foot infection and jaw bone infection, and are currently employed to study other osteomyelitic symptoms in many osseous tissues including long bones, vertebral column, clavicle, and sternum.

### Diabetic Foot Osteomyelitis

DFO is a severe form of infection in diabetic patients and can result in lower extremity amputation if left untended. Despite being a severe concern among diabetic patients, there are currently no universally acknowledged guidelines for DFO diagnosis or treatment. DFO is generally accompanied by elevated blood sugar concentration, compromised immunity, and concurrent vascular insufficiency (Lipsky, 1997; Markanday, 2014; Tong et al., 2015), and these complications facilitate the hematogenous or contiguous microbial infections, leading to severe inflammations. The DFO pathogenesis is mainly regulated by the DFO microbiome, which is however influenced by many factors such as demographic characteristics, personal hygiene, grade of severity, and antibiotic therapies (Jneid et al., 2017).

**FIGURE 1:**
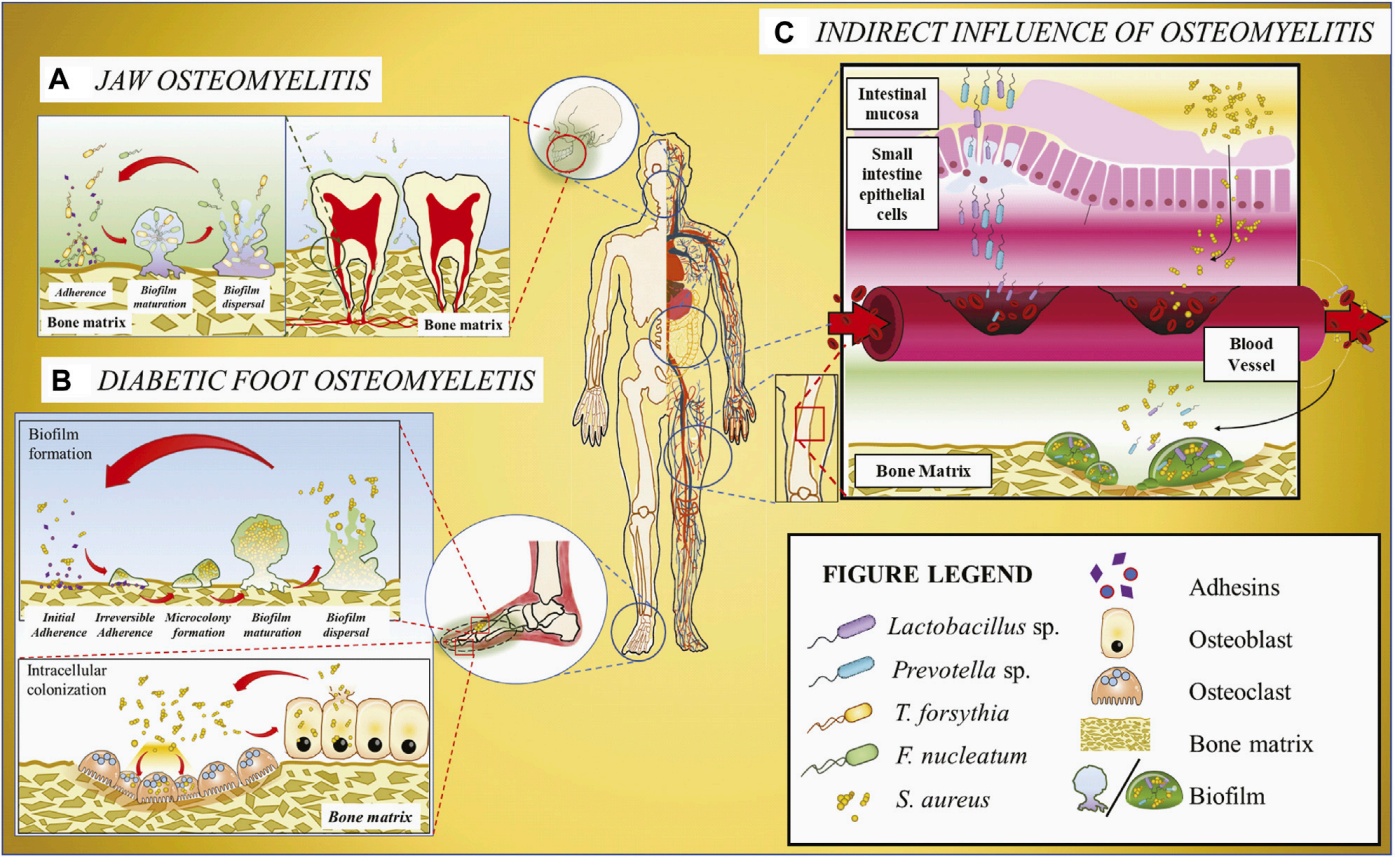
Schematic illustration of the host microbiome’s impacts on osteomyelitis. **(A)** Jaw osteomyelitis associated with two bacteria *T. forsythia* and *F. nucleatum* acts as a bridging bacterium to facilitate *T. forsythia* colonization and subsequent biofilm formation. **(B)** DFO involving *S. aureus*. As the most prevalent osteomyelitic pathogen, *S. aureus* may generate biofilm on the bone matrix or invade osteoclasts and osteoblasts mediated with adhesins, and the released bacteria from cells can be recovered to the original state. **(C)** Osteomyelitis or other osseous diseases associated with infectious microorganisms originating from gut microbiota. Some bacteria, e.g., *Lactobacillus* and *Prevotella*, translocate from gastrointestinal tract to the blood vessel across the gut epithelium barrier and then colonize in the bone matrix through the systematic circulation, ultimately resulting in osseous diseases.

Nevertheless, the DFO microbiota comprises a single dominant microbial species or a complex community infecting the bone. For instance, a study on infected osteomyelitic tissues showed that a single species infection by an anaerobic, non-motile, Gram-negative bacilli *Prevotella fusca* is positively correlated with the duration of diabetic foot infection (Zou et al., 2020). Other studies discovered that certain *Enterococcus faecalis* contribute to wound recovery, whereas *Staphylococcus aureus* infection can exacerbate an infected wound by triggering microbial pathogenesis (Jneid et al., 2018). The wound healing properties of *E. faecalis* are attributed to its probiotics status and its ability to help regulate the host immune responses (Franz et al., 2011). *S. aureus*, the most prevalent pathogen in osteomyelitic infections (Jagodzinski et al., 2009; Peltola et al., 2010; Hatzenbuehler and Pulling, 2011; Urish and Cassat, 2020), affects its host in several approaches (Figure 1).

*S. aureus* binds to the host tissues by expressing adhesins that facilitate the attachment to host extracellular matrix proteins such as collagen, fibrinogen, and fibronectin (Kdaa et al., 2020). Upon attachment, *S. aureus* evades the host immune cells and may form biofilms on the host tissue, increasing the microbial tolerance against any antimicrobial treatments. Previous studies have discovered *Staphylococcal* biofilm formation within chronic osteomyelitic bones (Gristina et al., 1985; Marrie and Costerton, 1985) and *in vitro* (O'Neill et al., 2007; Esteban et al., 2010). Additionally, commensal bacterial species are known to form multispecies biofilm, increasing the pathogenicity of the biofilm complex (Jneid et al., 2017). The commensal microbes within multispecies biofilm were documented in DFO specimens observed using SEM and/or CLSM-coupled PNA-FISH (Johani et al., 2019; Malone et al., 2019). The dynamic shift between the sessile and motile lifestyles of *S. aureus* in DFO tissues confers the pathogen resistance against non-surgical treatments, resulting in persistent chronic and acute infections.

Furthermore, *S. aureus* can infiltrate all types of osseous cells (osteoblasts, osteoclasts, and osteocytes) (Ellington et al., 2001; Klenerman, 2007; Reott et al., 2008; Mohamed et al., 2014; Yang et al., 2018) and then persist in a quiescent state, forming quasi-dormant small-colony variants (SCV) (Yang et al., 2018). This dormant lifestyle is less susceptible to antibiotics than the wild-type counterparts and can cause latent or recurrent infections upon release from these cells, potentially resulting in chronic infections (Proctor et al., 2006). Studies have demonstrated that the recovered SCV phenotypes were highly dynamic and could be rapidly reverted to the fully virulent wild-type form (Tuchscherr et al., 2011). These characteristics may explain the high infecting rate and recurrence of *S. aureus*-associated osteomyelitis.

Also, *S. aureus* produces a lot of factors that influence the host immune system. For example, immune evasion proteins SCIN and Efb help *S. aureus* evade the host immune system by inhibiting C3 convertase (Garcia et al., 2012), which belongs to the serine protease family and is necessary for innate immunity as a part of the complement system; chemotaxis inhibitory protein of *S. aureus* (CHIPS) inhibits neutrophil and monocyte chemotaxis toward C5a and formylated peptides by binding specifically to the C5aR and formylated peptide receptor (Postma et al., 2004); Staphylococcal protein A (SpA) can block antibody-mediated phagocytosis via binding Fcγ domain of Immunoglobulin G (Graille et al., 2000) or directly bind to osteoblasts, resulting in inhibition of osteoblastic proliferation and mineralization and even induction of apoptosis (Claro et al., 2011).

Studies of multispecies colonies have shown that certain coagulase-negative pathogenic *Staphylococci* such as *S. epidermidis* and *S. lugdunensis*, do not further exacerbate the wound severity compared to a single pathogen type infection (Yang et al., 2018). This unusual phenomenon agrees with the observation found in a study that showed reduced colonization of pathogenic *S. aureus* on the human nasal due to *S. lugdunensis* colonization (Zipperer et al., 2016). This inhibition of *S. aureus* colonization is hypothesized to be due to *S. lugdunensis* ability to produce lugdunin (thiazolidine-containing cyclic peptide antibiotics). The microbial interactions described above suggest intricate and subtle interactions between osteomyelitic microbes, functioning as both agonists and antagonists to regulate the host’s health.

### Jaw Osteomyelitis

Osteomyelitis of the jaw (i.e., maxilla and mandible) is a common disease found in patients suffering from head and neck infections (Baltensperger and Eyrich, 2009); however, there currently is no conclusive microbiome analysis of jaw osteomyelitis. There are some microbes specific to jaw osteomyelitis that have been identified (Table 1). One such example is that an *Escherichia coli* strain exhibiting multiple antibiotic resistances was isolated from bilateral maxillary osteomyelitis of a diabetic individual (PaDhiary et al., 2013). This strain presumptively enters the oral cavity through animal feces-contaminated water or food and then colonizes the jaw. Some soil-inhabited filamentous bacteria, *actinomyces*, were also discovered in maxilla osteomyelitis patients, and the entry portal is suggested to be from pulpal or periodontal infection (Gannepalli et al., 2015). *Actinomyces* are common commensals in the human gastrointestinal tract that may revert to be pathogenic upon infiltration of tissue layers or bones in this case. Other commonly found microbes in jaw osteomyelitic patients include *Cutibacterium acnes* (Park et al., 2017), a commensal strain of human skin and mucosal surface. The sequencing of *C. acnes* genome shows the presence of genes that are responsible for bacterial evasion from host immune system, biofilm formation, and resistance to clinical treatments such as multiple antibiotic treatment.

Similar to DFO, chronic osteomyelitis of the jaw (COMJ) pathogenesis is also associated with multispecies microbial communities. These microbial communities may exhibit antagonistic or synergistic interactions among the microbial community. A study of mandible or maxilla COMJ of twelve Brazilian patients showed three predominant commensal anaerobic strains (*Parvimonas micra*, *Staphylococcus* spp. and *Fusobacterium nucleatum*), indicative of the co-existence of these microbes resulting in the COMJ pathogenesis (Gaetti-Jardim Júnior et al., 2010).

Studies using NGS on COMJ provide vital information on the microbiome composition (Goda et al., 2014). It was discovered that the core microbiome comprises predominantly of the anaerobic microbes *Fusobacterium nucleatum* followed by *Tannerella* sp. and *Porphyromonas* sp. although the microbial populations from COMJ patient samples dramatically vary depending on the disease progression and the patient’s bone health. An *in vitro* study shows that *F. nucleatum* synergistically forms biofilms with *Tannerella forsythia* (Figure 1) (Sharma et al., 2005) dependent on surface contact rather than the bacterial biochemical cues; moreover, polymicrobial biofilms on a bone surface have been observed in an osteomyelitic jaw (Sedghizadeh et al., 2009). Hence, it is possible that *F. nucleatum* may also form biofilms with *T. forsythia* in COMJ. Other studies on *F. nucleatum* revealed that it acts an opportunistic pathogen in multispecies infections such as bacterial vaginosis (Citron, 2002), acute appendicitis (Swidsinski et al., 2011), and anaerobic bacteremia (Brook, 2010). *F. nucleatum* plays a crucial role in these infections by being a bridging bacterium to assist in the colonization of other bacteria, e.g., providing coaggregation conditions and an anaerobic environment for other anaerobes propagation. Thus, it is hypothesized that *F. nucleatum* plays a similar role in the pathogenesis and biofilm formation in the jaw bone, resulting in the development of COMJ. In addition, the multispecies biofilm of *F. nucleatum* with *P. gingivalis* and *T. forthysia* were found to induce severe periodontitis with massive bone resorption (Polak et al., 2009; Settem et al., 2012), suggesting that they may also synergistically form biofilms in COMJ.

## Osteomyelitis Indirectly Associated With Microbiome

The microbiome can indirectly influence osteomyelitis pathogenesis, in which the microbiota of non-osseous tissues produces biochemical signals that trigger the cells and microbes within the osteomyelitic tissue. In this section, we will discuss the impact of the gastrointestinal and serum-based microbiome on host osteomyelitis pathogenesis.

### Gastrointestinal Microbiome

The gastrointestinal tract (GIT) is the most densely microorganism-populated region of humans or mammals, giving rise to an ecosystem comprising commensal, symbiotic, and pathogenic microorganisms. The gastrointestinal microbiota alone outnumber their host’s genes by more than 100 times, whereas the changes of these populations are regulated by many factors including diet, lifestyle and the environment (Turnbaugh et al., 2009). Currently, the gut microbiota is closely correlated with human diseases, especially autoinflammatory diseases including asthma, arthritis, colitis, diabetes, and lupus (Bach, 2002; Chervonsky, 2010; Maslowski and Mackay, 2011; Bodkhe et al., 2019; Marietta et al., 2019), and modulation of the gut microbiome can be applied to treating some autoimmune diseases (Balakrishnan and Taneja, 2018). Although there are limited studies correlating the intestinal microbiome to osseous tissues, other studies focused on the host-microbe interactions have identified three main manners through which the gastrointestinal microbiome may influence distant organs (Hernandez et al., 2016), i.e., regulation of nutritional absorption, regulation of the immune system at the gut endothelium, and translocation of microbes and/or their metabolites across the endothelial barrier into the systemic circulation.

One such example was demonstrated in turkey poults fed with rye. The rye diet increases the *Lactobacillus* population in the intestinal microbiota while encouraging Enterobacteriaceae translocation, leading to significant reduction in bone strength and bone mineralization (Tellez et al., 2015). This study suggests that rye disrupts the epithelial tight junctions in the intestinal tract, causing infiltration of microbes into the systemic circulation and ultimately alterations of bone mineralization. Another example looked into the dietary intake in mice and its influence on the intestinal microbiota composition (Lukens et al., 2014). The mice fed with low-fat diets had enriched *Prevotella* populations and lower abundance of *Lactobacillus* compared to mice fed with normal diets. Mice fed with high-fat diets showed changes in intestinal microbiota that inhibit the osteomyelitis development in osteomyelitis-susceptible Pstpip2^cmo^ mice (Phillips et al., 2016). The Pstpip2^cmo^ mouse expresses a homozygous Leu98Pro missense mutation in the *Pombe* Cdc15 homologous protein PSTPIP2 (proline-serine-threonine phosphatase interacting protein 2), resulting in the increased susceptibility of the mice to develop autoinflammatory diseases, bone deformities, and elevated levels of IL-1β (Hartland, 2020). Further studies revealed that these gut microbiome alterations upregulate pro-IL-1β levels, suggesting that the gut microbiome can indirectly affect osteomyelitis via regulating pro-IL-1β levels in the circulatory system.

Additionally, studies have shown variation in GIT microbiota from rodents and humans of different genders, where these changes impact the host differently. Studies on rodents revealed that female B6 mice have higher abundance of *Lactobacillaceae* and *Bacteroides* compared to males; whereas female BALB/c mice have higher abundance of *Bifidobacteriaceae* than males (Elderman et al., 2018). Similarly, such changes in the microbiota are observed in human patients, where microbiota isolated from elderly women has lower abundance of *Bacteroidetes* than elderly men (Mueller et al., 2006; Dominianni et al., 2015). This gender specific variation of the GIT microbiota primarily results from the changes in the host biochemistry, where these alterations of the microbiome affect bone formation and are correlated to the increased susceptibility of patients to various osseous diseases (e.g., osteoporosis). This increased susceptibility is likely due to the microbiome influencing various immunological-related genes (Elderman et al., 2018) and the production of sex hormones (Menon et al., 2013). Thus far, it has been understood that the microbiome in different genders are considerably complex, encompassing the hormone level changes, T-cell activation, and modifications in cytokine production (Ibáñez et al., 2019).

The perturbation of the gut microbiome is also associated with a number of other bone and joint diseases. The monocolonization of the commensal *Lactobacillus bifidus* in interleukin-1 receptor antagonist-knockout mice result in the spontaneous development of autoimmune T-cell-mediated arthritis. This localization resulted in a faster onset of the disease compared to normal mice (Abdollahi-Roodsaz et al., 2008). The *L. bifidus*-triggered arthritis results from the imbalance of T_REG_-T_H_17 cell homeostasis through TLR2–TLR4 signaling. The presence of *Prevotella copri* in the gut was found to trigger the onset of untreated rheumatoid arthritis. This phenomenon was discovered through sequencing rheumatoid arthritis patient’s stool samples (Scher et al., 2013), and the onset development of rheumatoid arthritis is possibly due to *P. copri*’s ability to dominate the intestinal microbiota.

### Blood Microbiome

Healthy human blood is not as sterile as previously perceived since discovering a diversified microbiome in healthy human blood (Potgieter et al., 2015; Païssé et al., 2016). These blood-borne microorganisms infiltrate the human host through infected wounds or microbial translocation from the respiratory or intestinal epithelium. These microbes then circulate the human host until finding a suitable site for localization. When some microbes reach the bones, where the capillaries are abundant but tortuous thereby containing relatively slower bloodstream, these microbes readily accumulate and ultimately block the capillaries, leading to osteonecrosis and a series of inflammatory reactions. It should be noted that although some blood microbes in the blood might be dormant due to the host immune reactions or antibiotic treatments, they could however be resuscitated at suitable conditions (Panaiotov et al., 2018) and be potentially pathogenic.

The bacterial chondronecrosis with osteomyelitis (BCO) is a common cause of lameness in commercial broiler chickens worldwide and results in substantial economic loss. BCO pathogenesis was found to be correlated to the blood microbiome. Studies indicate that the microorganisms associated with BCO may originate from the intestinal or respiratory tract microbiota, which crosses the epithelium barrier and then enters the bloodstream (Wideman and Prisby, 2013). Chickens with BCO have an increased abundance of *Staphylococcus* sp., *Granulicatella* sp., and *Microbacterium* sp. compared to healthy chickens (Mandal et al., 2016). A study on osteomyelitis-associated genes indicates that chickens suffering from BCO showed downregulated level of Runt-related transcription factor (*RUNX2*) and secreted protein acidic and cysteine rich (*SPARC*) genes (Paludo et al., 2017). *RUNX2* is an important transcription factor that regulates the shape and differentiation of osteoblasts. Downregulation of *RUNX2* blocks osteoblastic and chondrocyte differentiations, ultimately impairing the ossification process (Ma et al., 2010). *SPARC* is a calcium-binding matricellular glycoprotein and is involved in bone development, repair, and tissue remodeling (Orlando et al., 2013). We hypothesize that the dysbiosis of the blood microbiome results in the downregulation of *RUNX2* and *SPARC* in BCO chickens, which further leads to osteomyelitis.

### Oral Microbiome

The oral cavity is the primary gateway to the human body, and the plethora of microorganisms (over 600 prokaryote species according to human oral microbiome database), whether colonizing or transiently inhabiting in the oral cavity, are very likely to translocate to different body sites. The human oral microbiome is the most extensively studied human microflora due to its relatively simple sampling process and strong correlation with common oral infectious diseases (e.g., dental caries and periodontitis). However, despite the extensive studies on the oral microbiome, there is much more to be explored as the microbiome network remains difficult to unravel due to the highly dynamic microbial compositions and complex interactions between microbe-host/microbe-microbe that impact the host biochemistry. Moreover, the variation of microbial distribution within the microenvironment of the oral cavity is also complex. For example, swabs from the center of the tongue exhibit more *Streptococcus salivarius* whereas the left outer part of the tongue is predominantly *Haemophilus parainfluenza* (Zeus et al., 2019). These changes in the microbiome composition influence the susceptibility of the host acquiring jaw osteomyelitis (discussed in *Jaw Osteomyelitis* section), providing an access for the oral microbiome to translocate to other parts of the human host. Despite these confounding factors, the oral microbiome may exhibit a possible role as modulatory target or biomarker in children and adolescents with chronic nonbacterial osteomyelitis (Zeus et al., 2021).

## Summary

Changes in the host-microbiome are known to affect osteomyelitis pathogenesis in direct and indirect manners. The direct interaction of osteomyelitic microbiota induces inflammation by forming single- or multi-species biofilms on the surface of the bones. Some of these microbes can also burrow into the host cells forming quasi-dormant small-colony variants (SCV). These dormant microbes are protected from the host immune system or antibiotic treatments and shift to a planktonic lifestyle when there is a change in the host biochemistry. The microbiota from other niches indirectly influences osteomyelitis by altering the biochemical signals that regulate the host immune responses. Certain microbiota members can infiltrate into the host systemic circulation and then generate microbial embolism, causing tissue necrosis and chronic diseases such as bacterial chondronecrosis with osteomyelitis. Thus, it can be concluded that the various microbiomes within the patient influence the severity of the osteomyelitis. Further understanding of these microbial populations’ roles would be needed to improve the treatment of osteomyelitis and encourage rapid patient recovery by regulating the host microbiome.

## Abbreviations

BCO, bacterial chondronecrosis with osteomyelitis; COMJ, chronic osteomyelitis of the jaw; CLSM, confocal laser scanning microscope; GIT, gastrointestinal tract; SCV, small-colony variants; SEM, scanning electron microscopy; PNA-FISH, peptide nucleic acid fluorescent in situ hybridization.
